# Saroglitazar improved hepatic steatosis and fibrosis by modulating inflammatory cytokines and adiponectin in an animal model of non-alcoholic steatohepatitis

**DOI:** 10.1186/s40360-021-00524-8

**Published:** 2021-10-01

**Authors:** Rasoul Akbari, Tahereh Behdarvand, Reza Afarin, Hamid Yaghooti, Mohammad Taha Jalali, Narges Mohammadtaghvaei

**Affiliations:** 1 Hyperlipidemia Research Center, Ahvaz Jundishapur University of Medical Sciences, Ahvaz, Iran; 2 Department of Laboratory Sciences, School of Allied Medical Sciences, Ahvaz Jundishapur University of Medical Sciences, Ahvaz, Iran

**Keywords:** Saroglitazar, NASH, PPAR- α/γ, TNF- α, TGF-β, MCP-1, IL-6

## Abstract

**Background:**

Non-alcoholic fatty liver disease (NAFLD) and non-alcoholic steatohepatitis (NASH) have become significant global health concerns. In the present study, we aimed to investigate the effects of saroglitazar, a dual PPARα/γ agonist, fenofibrate, a PPAR-α agonist, and pioglitazone, a PPAR-γ agonist on an animal model of NASH.

**Methods:**

Male Wistar rats were fed a high-fat (HF) emulsion via gavage for 7 weeks to induce NASH. The HF-treated rats were grouped into four groups to receive saroglitazar, pioglitazone, fenofibrate, or vehicle. We measured body and liver weight, liver enzymes, serum levels of adiponectin and leptin. We also performed histopathological examinations and gene expression analysis of interleukin 6 (IL-6), tumor necrosis factor-alpha (TNF- α), transforming growth factor-beta (TGF-β), and monocyte chemoattractant protein 1 (MCP-1).

**Results:**

Body weight was markedly normalized by both saroglitazar and fenofibrate, while the liver index only decreased significantly with saroglitazar. Saroglitazar corrected ALT, AST, leptin, and adiponectin levels better than pioglitazone and fenofibrate. All PPAR agonists significantly attenuated the upregulation of the proinflammatory and TGF-β genes, which correlated with the improved steatosis, inflammation of liver tissue, and fibrotic lesions.

**Conclusions:**

As documented by our results, the dual activation of PPARα/γ by saroglitazar could effectively improve steatosis, fibrosis, and aspects of necro-inflammation in the HF-induced NASH model more than fenofibrate and pioglitazone, and it can be more beneficial in the management of NASH.

## Background

Non-alcoholic fatty liver disease (NAFLD), a metabolic liver disorder, is globally the most common liver disease. Progression to its dangerous form, non-alcoholic steatohepatitis (NASH), is becoming increasingly prevalent [1]. Hepatic steatosis in NAFLD is characterized by a considerable accumulation of fat droplets within hepatocytes. Consequent hepatic inflammation and hepatocellular injury with or without hepatic fibrosis lead to the advanced stages of the disease termed NASH. According to the multi-hit hypothesis, multiple factors predispose to the development of NAFLD and NASH [2]. Among them, inflammation, oxidative stress, endoplasmic reticulum stress, altered lipid metabolism, insulin resistance, altered production of adipokines and cytokines have prominent roles in the disease [2–4]. It is well established that adipose tissue plays a pivotal role in developing NASH through the secretion of adipokines such as leptin, adiponectin, and inflammatory cytokines [5]. Adipose tissue expansion disturbs the balance between leptin and adiponectin by decreasing adiponectin and increasing leptin, leading to obesity, insulin resistance, and hepatic steatosis [5–7]. Tumor necrosis factor (TNF)-α is a proinflammatory cytokine secreted by adipose tissue, hepatocytes, and Kupffer cells in the liver. It plays an essential role in developing hepatic steatosis and mediates hepatic inflammation, oxidative stress, and apoptosis or necrosis of hepatocytes [8, 9]. Monocyte chemotactic protein-1 (MCP-1), transforming growth factor (TGF)-β, and interleukin-6 (IL-6) are proinflammatory cytokines produced by resident macrophages in hepatocytes, which are dependent on TNF-α acting as an upstream mediator [10, 11]. TGF-β1 is the main liver isoform that plays a critical role in hepatic fibrosis by mediating the activation of stellate cells and their production of extracellular matrix proteins [12, 13]. Also, MCP-1 is a potent chemokine mainly secreted by macrophages and hepatic stellate cells [14]. MCP-1, through multiple mechanisms, contributes to the pathogenesis of NASH, including hepatic lipid accumulation, insulin resistance, and coordinates leukocyte recruitment to the liver [15]. The role of IL-6 in the liver is very complex. IL-6 sensitize the liver to injury, stimulate hepatocyte apoptosis, induce insulin resistance, and participates in NASH development [13].

Peroxisome proliferator-activated receptor alpha/gamma (PPARα/γ) receptors modulate glucose and lipid metabolism in the liver, adipose tissues, and muscles. PPARα/γ dual agonists have shown good potentials to treat metabolic diseases [16]. The activation of PPARα stimulates fat consumption by inducing the expression of genes associated with beta-oxidation, resulting in improved hyperlipidemia. PPARγ, on the other hand, promotes the mobilization of fatty acid into adipocytes by encouraging adipogenesis and upregulating the expression of genes involved in fatty acid transport [17]. In addition, activation of PPARα increases adiponectin receptors in the adipose tissue and decreases MCP-1 expression in adipose tissue and macrophages [18]. Since PPARγ agonists induce storage of fatty acids and glucose rather than consumption in the cell, the dual activation of PPARα and PPARγ is proposed to be more beneficial in managing metabolic diseases such as dyslipidemia of diabetes and fatty liver.

In the present study, we investigated the comparative effects of saroglitazar (SARO), a dual PPARα/γ agonist, versus fenofibrate (FENO) and pioglitazone (PIO) as separate α and γ agonist on the adipose tissue factors and inflammatory components of NASH, developed in rats using a high-fat formulation.

## Materials and methods

### Preparation of the high-fat emulsion

The composition of the high-fat emulsion used to induce NASH is shown in Table 1. It provided 77% of its energy from fat, 9% from carbohydrates, and 14% from whole milk powder as a source of protein. We prepared the high-fat emulsion as described by Yuhong Zou et al. [19]. This emulsion was stored at 4 °C and was thoroughly mixed and heated in a 42 °C water bath daily before use.

**Table 1:** The composition of macronutrients and caloric content of the high-fat emulsion

Components of high fat emulsion	Amount
**Corn oil (g)**	**400**
**Saccharose (g)**	**150**
**Total milk powder (g)**	**80**
**Cholesterol (g)**	**100**
**Sodium deoxycholate (g)**	**10**
**Tween 80 (g)**	**36.4**
**Propylene glycol (g)**	**31.1**
**Vitamin mixture (g)**	**2.5**
**Cooking salt (g)**	**10**
**Mineral mixture (g)**	**1.5**
**Distilled water (ml)**	**300**
**Total energy (kcal/l)**	**4342**

### Chemicals

Saroglitazar was supplied by Cadila Healthcare Limited, Ahmedabad, India. Both fenofibrate and pioglitazone were purchased from the Abidi Pharmaceutical Company, Tehran, Iran. The drugs were suspended in 0.5% sodium carboxymethyl cellulose solution (CMC) for administration.

### Animals and experimental design

Adult male Wistar rats, weighing 180–200 g, were purchased from the Experimental Animal Center, Ahvaz Jundishapur University of Medical Sciences, and were quarantined for 1 week before the experiments started. Animals were kept in comfortable cages under controlled environmental conditions at a room temperature of 25 ± 3 °C with 55 ± 8% humidity under 12-h light-dark cycles. All experiments on laboratory animals were approved by the University Ethics Committee and performed according to the regulations for using and caring for experimental animals at Ahvaz Jundishapur University of Medical Sciences.

At first, 45 rats were randomly divided into two groups: the normal control (NC) group (*n* = 9) and the high-fat emulsion (HF) group (*n* = 36). The standard diet and water were available to all animals during the study. Rats in the high-fat emulsion group were orally given the high-fat emulsion (10 ml/kg) daily via gavage for 7 weeks. Additionally, they were allowed free access to drinking water containing 18% saccharose to induce a more characteristic NASH. During the seventh week, to ensure the development of NAFLD/NASH, two NC and four HF group rats were randomly sacrificed. Their livers were sent to our laboratory for pathological examinations. After confirming the successful model, pharmacological treatments were carried out separately from the 8th week for 6 weeks. For this purpose, animals in the HF group were randomly divided into groups I to IV (using a random number table) as follow; Group I: control HF that received equal volumes of 0.5% CMC solution (drug vehicle), II: high-fat emulsion plus fenofibrate 100 mg/kg body weight (HF + FENO group), III: high-fat emulsion plus pioglitazone 30 mg/kg body weight (HF + PIO group), IV:high-fat emulsion plus Saroglitazar 3 mg/kg body weight (HF + SARO group). After a high dose ketamine-xylazine injection at the end of treatments, we sacrificed 15 h fasting rats. Blood samples were drawn from the aorta ventralis. After dissecting the livers, they were washed with normal cold saline and dried on sterile filter papers. They were immediately weighed to calculate liver index (liver weight/body weight × 100). Each liver was cut into pieces, and aliquots of the liver tissue were snap-frozen in liquid nitrogen at − 180 °C for further gene expression analysis. A larger piece of liver was immersed in a 10% formalin solution for histopathological examination.

### Biochemical measurements

Levels of liver enzymes in serum, including alanine aminotransferase (ALT) and aspartate aminotransferase (AST), were measured using corresponding assay kits by the Roche 6000 auto-analyzer. Leptin and adiponectin concentrations were determined using enzyme-linked immunoassay kits (DRG Diagnostics, Marburg, Germany).

### Gene expression analysis

Gene expression of IL-6, TNF-α, TGF-β, and MCP-1 was evaluated by real-time PCR technique. For this purpose, total RNA was isolated from the frozen liver samples with the FastPure™ RNA Kit from Takara Bio (Otsu, Japan) according to the manufacturer’s protocol. The reverse transcription was performed using PrimeScript RT reagent Kit (Takara Bio, Otsu, Japan). Real-time PCR was carried out using SYBR Green Premix Ex Taq™ mix (Takara Bio, Otsu, Japan) and QuantStudio™ 3 Real-Time PCR System (ABI Applied Biosystems). The expression level of the target mRNA was normalized against β-actin expression as an internal standard. At the end of the real-time PCR procedure, relative quantification was performed with the Applied Biosystem software. The primer sequences used for the real-time PCR reactions are shown in Table 2.

**Table 2:** Primer pairs sequences

Gene	Forward primer	Reverse primer
**TNF- α**	5′-ACCACGCTCTTCTGTCTACTG-3′	5′-CTTGGTGGTTTGCTACGAC-3′
**IL- 6**	5′- TGATGGATGCTTCCAAACTG-3′	5′- GAGCATTGGAAGTTG GGGTA-3′
**TGF- β**	5′-CAAAGACATCACACACAGTA-3′	5′-GGTGTTGAGCCCTTTCCAGG-3′
**MCP1**	5′-GTGCTGACCCCAATAAGGAA −3′	5′-TGAGGTGGTTGTGGAAAAGA-3′
**β-Actin**	5′-CCCATCTATGAGGGTTACGC-3′	5′-TTTAATGTCACGCACGATTTC-3′

### Histopathological evaluations

Pieces of liver tissue were removed from the formalin solution, and following dehydration with gradient alcohol, they were embedded in paraffin wax. Sections of 6–7 μm thickness were cut and stained with hematoxylin-eosin (HE) and Masson’s trichrome in two parts to investigate hepatic steatosis, inflammation, necrosis, and fibrosis. Histopathological changes were evaluated by an experienced liver pathologist who was blind to the interventions. The severity of steatosis, inflammation and fibrosis were scored and graded according to the NASH activity score (NAS), described by Kleiner, D. E, et al. and Liang, W, et al. [20, 21].

### Statistical analysis

The data were statistically analyzed with the one-way analysis of variance (ANOVA) followed by Tukey’s post hoc test for multiple comparisons using GraphPad Prism version 8.0.2 for Windows (GraphPad Software, La Jolla, CA, USA). Results were considered significant at *P*-value < 0.05.

## Results

### Changes in body weight and liver index in response to treatments

**Fig. 1:**
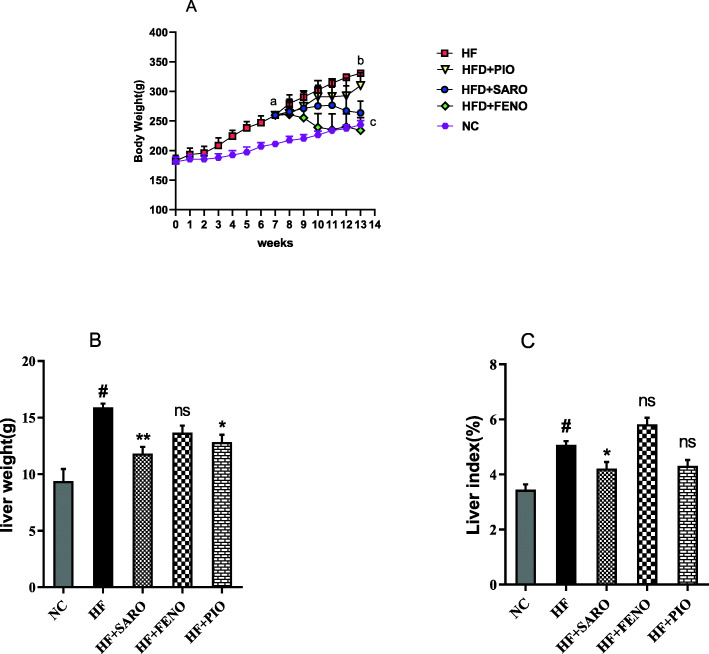
Effect of saroglitazar, fenofibrate, and pioglitazone on the body weight (A), liver weight (B), and percentage of the liver index (C) in the HF-induced NASH model. Values are expressed as mean ± SD (*n* = 7). NC = normal control, HF = high-fat emulsion, SARO = Saroglitazar, FENO=Fenofibrate, PIO=Pioglitazone. **a** and **b** indicate Significant difference against NC after 7 and 13 weeks, **c** indicates significant differences between NC, HF + SARO, and HF + FENO vs. HF, *p* < 0.001.*p < 0.05; **p < 0.01; and ns (nonsignificant) vs HF. # indicates significance vs. NC, *p <* 0.001

As shown in Fig. 1, A, there were no significant differences in body weights at the beginning of the experiment. Rats fed with the high-fat emulsion for 13 weeks showed significantly increased body weight (*p* < 0.001), liver weight (*p <* 0.001), and liver index (*p* < 0.01) in comparison to the NC group. After the next 6 weeks of treatment, the increase in body weight was markedly normalized by both saroglitazar and fenofibrate (*p <* 0.01). In contrast, treatment with pioglitazone did not correct the body weight compared to the HF group. Also, treatment with saroglitazar and pioglitazone significantly reduced the liver weight compared to the HF group (*p* < 0.01 and *p* < 0.05, respectively, Fig. 1B). Figure 1C shows that saroglitazar treatment for 6 weeks decreased liver index by 17% compared to the HF group (*p <* 0.05). In contrast, treatment with fenofibrate increased liver index, but the increase was not statistically significant.

### Saroglitazar corrected the elevated liver enzymes and adipokines in the NASH model

ALT and AST as biomarkers of liver injury were measurements in this study. Feeding rats with the high-fat emulsion led to marked elevations in ALT and AST levels in serum compared to the rats in the NC group (*p* < 0.001, Table 3). The PPAR agonists differentially reversed the injurious effect of high-fat emulsion and decreased ALT and AST to different levels (*p* < 0.05). Saroglitazar showed better efficacy in reducing and normalizing ALT and AST levels than pioglitazone and fenofibrate.

**Table 3:** Biochemical parameters of rats in different groups

Parameters	NC	HF	HF + SARO	HF + FENO	HF + PIO
ALT (IU/l)	33.47 ± 4.93	69.12 ± 7.33#	37.81 ± 4.51 ***	53.26 ± 8.61**	51.48 ± 9.71**
AST (IU/l)	42.29 ± 4.15	103.91 ± 16.75#	39.08 ± 12.56 ***	66.21 ± 5.01***	84.73 ± 5.37 *
Leptin (ng/ml)	1.41 ± 0.23	3.12 ± 0.65#	1.86 ± 0.53 ***	2.37 ± 0.41*	2.19 ± 0.31**
Adiponectin (mg/ml)	5.61 ± 0.78	1.55 ± 0.46#	4.70 ± 0.99 ***	3.95 ± 0.83 ***	3.39 ± 1.03 *

Values are expressed as the mean ± S.D. of 7 rats. Between group comparisons was tested by ANOVA followed by Tukey Kramer multiple comparisons test. *NC* normal control, *HF* high-fat emulsion, *SARO* Saroglitazar, *FENO* Fenofibrate, *PIO* Pioglitazone.^*^p < 0.05; ^**^p < 0.01; ^***^p < 0.001; and ns (nonsignificant) vs. HF. ^#^ *p* < 0.001 vs. NC

As shown in Table 3, feeding rats with the high-fat emulsion caused an almost 2.1-fold increase in leptin level and a 3.6-fold decrease in adiponectin level in serum compared to those in the NC group. All drugs successfully reversed the increase in leptin level (SARO; *p* < 0.001, FENO; *p* < 0.05, PIO; *p* < 0.01). Also, treatment with saroglitazar (*p <* 0.001), fenofibrate (*p <* 0.001) and, pioglitazone (*p <* 0.05) significantly enhanced adiponectin level as compared to the HF group. Saroglitazar showed a higher effect on correcting the levels of adipokines.

### Saroglitazar attenuated the expression of proinflammatory and profibrogenic mRNAs

**Fig. 2:**
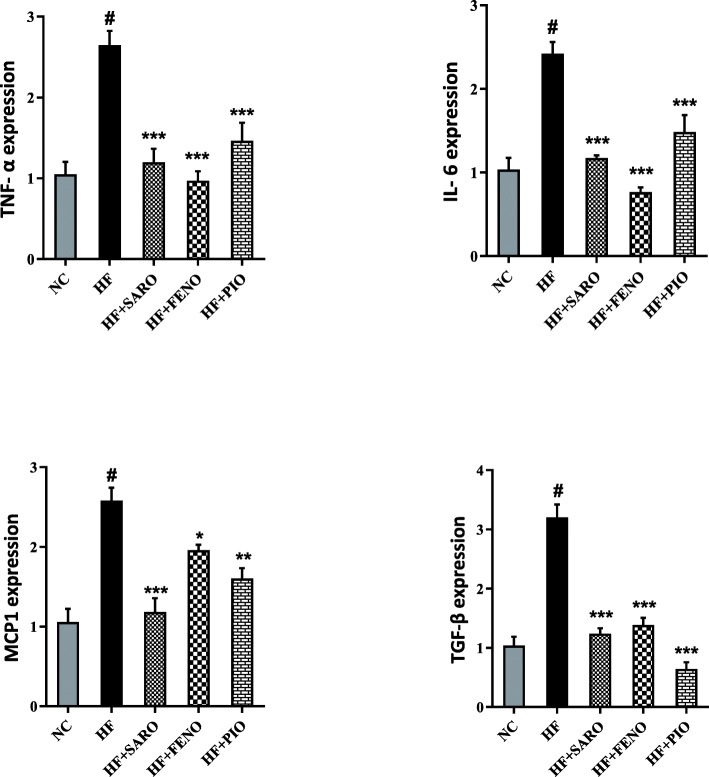
Gene expression levels of proinflammatory cytokines and TGF-β in response to treatment with PPAR agonists. Hepatic mRNA levels were determined using quantitative real-time PCR and normalized to β-actin mRNA expression. Values are expressed as the mean ± S.D. of fold changes relative to the NC. Between-group, comparisons were tested by ANOVA followed by Tukey Kramer multiple comparisons test. TNF- α, tumor necrosis factor-alpha; IL-6, interleukin 6; MCP-1, monocyte chemoattractant protein 1; TGF-β, transforming growth factor-beta. ^*^p < 0.05; ^**^p < 0.01; ^***^p < 0.001; and ns (nonsignificant) vs. HF. ^#^ *p <* 0.001 vs. NC

We found a marked increase in hepatic mRNA expression of proinflammatory and pro-fibrogenic genes, including TNFα, IL-6, MCP1, and TGF-β in the qPCR analysis of gene expression in the HF group compared to the NC group (*p <* 0.001 for all, Fig. 2). Treatment with saroglitazar, fenofibrate, and pioglitazone significantly decreased the expression of proinflammatory genes (*p* < 0.001). The mRNAs level of TGF-β as a fibrogenic mediator and MCP1 were increased 2.5-fold and 3.2- fold, respectively, following high-fat emulsion feeding (*p* < 0.001). Our results showed that saroglitazar decreased the elevated MCP 1 mRNA more than fenofibrate and pioglitazone (*p <* 0.001 vs. *p* < 0.05 and *p* < 0.01, respectively). In addition, all the PPAR agonists could significantly decrease the upregulated mRNA of TGF-β following the high-fat regime (*p <* 0.001). The effect of pioglitazone was the highest in this regard.

### Histopathological findings

**Fig. 3:**
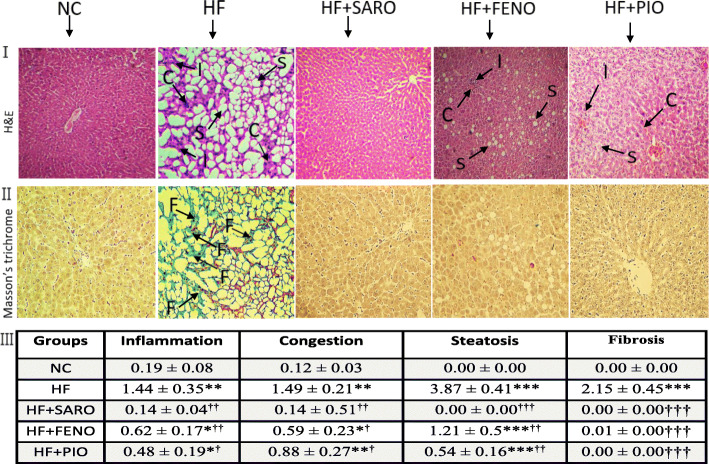
Histopathological evaluation of the NASH model following treatment with PPAR agonists. Upper, representative images of hematoxylin-eosin and Masson Trichrome stained liver tissue sections with 100X magnification in different groups at the end of treatments. C: congestion; I: inflammation; S: steatosis; F: fibrosis. Lower, mean ± S.D. of scores measured for steatosis, congestion, and inflammation after H&E staining and for fibrosis, deposition of collagen was evaluated using the Masson’s trichrome staining (*n =* 7). *p < 0.05, **p < 0.01, ***p < 0.001 vs. NC; † *p <* 0.05, †† *p <* 0.01, ††† *p <* 0.001 vs. HF

As illustrated in Fig. 3, microscopic examination of liver sections of rats in the HF group showed congestion, grade 3 hepatocellular steatosis, inflammation, and extensive fibrosis of stage 3 compared to the liver sections of the control rats with no indication of fat accumulation, inflammation, or fibrosis. These findings were confirmed by the H&E staining and Masson’s trichrome staining (Fig. 3, I and II, respectively). Saroglitazar administration for 6 weeks significantly improved fatty appearance, lobular inflammation, hepatocellular ballooning of liver tissue, and decreased fibrotic lesions. These parameters were also alleviated by fenofibrate and pioglitazone treatments comparable to saroglitazar administration (Fig. 3, I, II, and III). However, saroglitazar represented better scores in improving histopathological lesions than fenofibrate and pioglitazone (Fig. 3, III).

## Discussion

The mechanism of development and progression of NAFLD/NASH is multifactorial and complex. According to the “two-hit hypothesis,” the first event is fat accumulation in the liver. The second hit is activating inflammatory pathways, oxidative stress, and fibrogenesis [22]. Drugs that target the individual components of NASH pathogenesis have not been very effective so far. Efforts to find an ideal drug that can manage NASH’s multifaceted condition have not been successful. Since PPAR-α and PPAR-γ agonists can improve insulin resistance, liver steatosis, oxidative stress, inflammation, adipocytokines secretion, and fibrosis, saroglitazar as a dual PPAR-α/γ agonist, seems to be a rational choice in this condition. Our results showed that saroglitazar alleviated the NASH damages in our model better than fenofibrate and pioglitazone.

PPARs have crucial roles in regulating several biological processes associated with NASH, including inflammation, lipid and glucose homeostasis, and fibrosis [23, 24]. Our results showed that treatments with all three PPAR agonists could significantly attenuate the increased inflammatory cytokines and TGF-β as a fibrotic marker in our NASH model. As the primary mechanism, PPARs inhibit nuclear factor-kappa B (NF-κB) activation and suppress its downstream inflammatory genes such as TNF-α and IL-6 [25]. TNF-α plays a pivotal role in the progression of NAFLD to NASH through the induction of regulatory molecules associated with lipid and glucose metabolism, inflammatory cytokines, and fibrosis in the hepatocytes. TNF-α also directly induces MCP1 and TGF-β expression as essential mediators in inflammation and fibrogenesis [10]. Earlier studies have shown PPARγ agonists to inhibit TNF-α and its associated cytokines. PPARγ agonists decreased TGF-β in animal models of NASH and cholestatic fibrosis [26]. PPARα and PPARγ agonists have the potential to correct non-alcoholic steatohepatitis damages. Thus, co-activation of PPARα and PPARγ using a dual PPARα/γ agonist such as saroglitazar could be a reasonable approach to ameliorative NASH. In line with these findings recently Kumar et al. have demonstrated applying saroglitazar in a Diet induced animal model of NASH reduces hepatic ER stress, inflammation and fibrogenic signaling [27]. While it has been shown PPAR agonists differently affect inflammation and fibrosis, a fundamental question is how the new dual PPARα/γ agonist is different from the more common α and γ agonists, especially concerning the adipokines in relation to inflammation and fibrosis in the context of NASH, which has not been studied before. Here we compare modulating effects of saroglitazar with fenofibrate a PPARα agonist and pioglitazone a PPARγ agonist. Our results confirmed that the dual PPARα/γ agonist saroglitazar improved experimental NASH’s biochemical and histopathological complications better than fenofibrate and pioglitazone.

Leptin and adiponectin are mainly secreted from adipose tissue and play essential roles in developing and progressing fatty liver disease. In obesity, leptin resistance augments the production and secretion of leptin [28]. Elevated leptin levels activate Kupffer cells and liver stellate cells, which give rise to the inflammatory processes in the liver [29], In contrast, adiponectin exerts anti-inflammatory properties in the liver and improves hepatic and peripheral insulin resistance. Adiponectin inhibits hepatic production of proinflammatory cytokines such as TNF-α, IL-6 by suppressing NF-κB activity [30]. To the best of our knowledge, this is the first study that suggested saroglitazar may exert its beneficial effects on hepatic steatosis through modulating leptin and adiponectin levels in the model of NASH.

Our findings confirmed that PPARα and PPARγ activation favorably modified the adipokines by decreasing leptin and increasing adiponectin levels. In addition, PPAR-α activation can upregulate adiponectin receptor1 expression in visceral adipose tissue, which gives rise to enhanced adiponectin effects [18, 31]. Co-activation of PPARα and PPARγ by saroglitazar treatment presented a better adipokine profile than fenofibrate and pioglitazone alone. It has been shown that correcting leptin and adiponectin levels can prevent profibrogenic responses by inhibiting TGF-β in experimental models of NASH and chemicals-induced liver injury [32, 33]. Similarly, we showed that correcting the adipokines levels in response to PPAR agonists correlated well with improving the fibrosis scores at the end of treatments.

Adiponectin and TNF-α expression have a significant inverse correlation in adipose tissue. Injection of adiponectin suppressed TNF-α expression and reduced the plasma level of TNF-α that led to the prevention of fat accumulation and inflammation in several mouse models of chronic liver injury [34, 35]. On the other hand, treatment with TNF-α suppressed the expression of adiponectin by inhibiting PPAR-γ in adipocytes [36]. Therefore, adiponectin is known to act as a potent anti-inflammatory and antifibrotic cytokine and a suitable target for treating steatohepatitis, while leptin exerts an inverse role [37–39]. Our results showed that saroglitazar effectively targeted adiponectin and leptin expression to suppress inflammatory and fibrogenic cytokines such as TNF-α, IL-6, MCP1, and TGF-β in the NASH model.

The H&E and Masson’s trichrome staining and Quantitative histopathological assessments showed strong antisteatotic and antifibrotic effects of saroglitazar. This effect was revealed by the disappearance of lipid droplets and collagen deposits in liver tissue. Separate PPARα- and PPARγ agonists could not provide the improvement provided by saroglitazar. This finding follows an earlier study that showed saroglitazar improved steatosis, while fenofibrate did not decrease hepatic lipid droplets in Zucker fa/fa rats [40]. In the present study, AST, and ALT as serum markers of NASH were significant reduced after 6 weeks of Saroglitazar treatment more pronounced as compared to fenofibrate or pioglitazone. These findings are consistent with clinical studies that were confirmed saroglitazar reduced the levels of these liver enzymes in patients with NAFLD and diabetic dyslipidemia [41, 42]. Fenofibrate, a selective PPARα agonist, corrects hyperlipidemia by enhancing β-oxidation and causes a reduction in body weight. Pioglitazone is a synthetic PPARγ agonist used to lower blood glucose levels and lipotoxicity by modifying insulin resistance. Pioglitazone regulates the expression of lipid metabolism genes, stimulates glucose uptake in adipose tissue, and induces adipocyte differentiation [43]. However, since PPARγ agonists enhance the deposition of fatty acids and glucose rather than oxidation in the cell, there have been concerns regarding their side effects, such as body weight gain and clinical application in conditions such as the fatty liver. Treatment with pioglitazone in our study caused a significant increase in body weight following previous findings that treatment with TZDs is associated with body weight increase in obese animals and type 2 diabetic patients [41, 44–47]. In contrast, fenofibrate treatment completely normalized body weight compared to the HF group and increased the liver index, similar to the previous findings [48]. Co-treatment of fenofibrate and pioglitazone corrected the enlargement of the liver index [49]. Likewise, our results showed that saroglitazar treatment normalized body weight, liver weight, and liver index. Consistent with our findings, clinical studies also were demonstrated that taking Saroglitazar 4 mg improved glycemic index, lipid profile, and weight loss in patients with NAFLD and type 2 diabetes [41, 42]. Therefore, as documented by our results, the dual activation of PPARα/γ is proposed to be more beneficial in NASH management.

In this study, we did not evaluate the protein expression of IL-6, TNF- α, TGF-β, and MCP1 to confirm the mRNA expression of these molecules in the liver. However, this study primarily focused on the effects of saroglitazar on genetic and phenotypic features of NASH, which is supported by histopathological findings and the expression of inflammatory genes linked with NASH. We also evaluated circulating levels of leptin and adiponectin known as markers for the diagnosis and therapy in response to saroglitazar to demonstrate its anti-inflammatory effects in the context of NASH. Our data will help drive future studies to identify molecular mechanisms by which saroglitazar improves NAFLD/ NASH.

## Conclusions

Our results suggest that the dual activation of PPARα and PPARγ by saroglitazar can effectively improve steatosis, fibrosis, and aspects of necro-inflammation in the HF-induced NASH model better than fenofibrate and pioglitazone. This approach may resolve drawbacks and side effects associated with the separate administration of PPARα or PPARγ agonists.

## Data Availability

The datasets used and/or analyzed during the current study are available from the corresponding author on reasonable request.

## Abbreviations

NAFLD: Non-alcoholic fatty liver disease
NASH: Non-alcoholic steatohepatitis
HF: High fat
PPARα: Peroxisome Proliferator-Activated Receptor α
PPAR γ: Peroxisome Proliferator-Activated Receptor γ
NF-κB: Nuclear factor-kappa B
IL-6: Interleukin 6
TNF- α: Tumor necrosis factor alpha
TGF-β: Transforming growth factor beta
MCP-1: Monocyte chemoattractant protein 1
CMC: Carboxymethyl cellulose
SARO: Saroglitazar
PIO: Pioglitazone
FENO: Fenofibrate
